# EPO activates PI3K-IKKα-CDK1 signaling pathway to promote the proliferation of Glial Cells under hypoxia environment

**DOI:** 10.1590/1678-4685-GMB-2021-0249

**Published:** 2022-02-11

**Authors:** Gejile Hu, Ting Wang, Chunjie Ma

**Affiliations:** 1 Beijing University of Chinese Medicine, School of Traditional Chinese Medicine, Beijing, China.; 2 Hospital of Inner Mengolia Medical University, Hohhot, Inner Mengolia, China.; 3 Inner Mongolia University, School of Life Sciences, Hohhot, Inner Mengolia, China.; 4 Inner Mongolia Medical University, School of Traditional Chinese Medicine, Hohhot, Inner Mengolia, China.

**Keywords:** EPO, glial cell, hypoxia, signaling pathway, neuroprotection

## Abstract

Erythropoietin (EPO), supports the function and survival of neurons through astrocytes and has a protective role in neonatal asphyxia brain injury; yet, its mechanism of action remains unclear. As a neuroprotective factor, EPO is also used in the treatment of various diseases, such as neurodegenerative diseases, Parkinson’s disease, traumatic brain injury, by decreasing inflammatory reaction, resisting apoptosis, and lowering oxidative stress. The aim of this study was to examine the effect and mechanism of EPO on promoting human brain glial cell proliferation under hypoxia in vitro. Under CoC1_2_-induced hypoxia, after adding EPO, high-throughput sequencing was used to screen out meaningful up-regulated and significant differentially expressed genes PI3K, IKKα CDK1 related to proliferation, and make further verification by qPCR and western blotting. Under hypoxia, EPO promoted cell proliferation and the expression of PI3K while this effect was inhibited (along with a decrease of downstream genes IKKα and CDK1 decreased) after adding PI3K inhibitor to cell culture. EPO can promote cell proliferation and CDK1 expression, while after inhibiting CDK1 expression, the promotion of EPO on cell proliferation was eliminated. These data proved that EPO promotes the proliferation of U251 cells by activating the PI3K-IKKα-CDK1 signaling pathway under CoC1_2_-induced hypoxia.

## Introduction

Neonatal asphyxia refers to a critical pathological state during which gas exchange between maternal and fetal blood flow is blocked, resulting in hypoxia and the inability to establish and maintain normal respiration after birth (Ahearne et al., 2016). The brain is a commonly damaged organ of neonatal asphyxia (Baburamani et al., 2015). Asphyxia can induce brain hypoxia, causing edema and cell apoptosis, leading to necrosis of brain tissue and eventually brain injury (Wang et al., 2015). Neonatal asphyxia often leads to neonatal hypoxic-ischemic encephalopathy, cerebral palsy, intellectual disability, and neurovascular injury, which are a common cause of neonatal death worldwide (Simiyu et al., 2017). 

Astrocytes participate in the regulation of neuronal function in the central nervous system and support the function and survival of neurons through various mechanisms (Toriuchi *et al.*, 2020). Activation of astrocytes can prevent cerebrovascular dysfunction after asphyxia and has a neuroprotective effect on neonatal asphyxia brain injury. However, the role of glial cells in neonatal asphyxia is complex and has not yet been fully understood (Parfenova et al., 2018).

Erythropoietin (EPO) is a sialic acid-containing glycoprotein hormone that binds to its receptor (EPOR) expressed on glial cells (Nagai et al., 2001). As a neuroprotective factor, EPO is used in the treatment of various diseases, such as neurodegenerative diseases (Merelli et al., 2015; Maiese, 2016b), epilepsy (Castaneda-Arellano et al., 2014), Parkinson’s disease (Punnonen et al., 2015), traumatic brain injury (Maiese, 2016a), diabetic neuropathy (Javed et al., 2015), etc. Some studies have suggested that EPO can reduce the damage caused by neonatal asphyxia (Alexander et al., 2012), improve neovascularization (Kolusari et al., 2018), and reduce the damage of the central nervous system by decreasing inflammatory reaction, resisting apoptosis (Villa et al., 2003), and lowering oxidative stress and peroxide level (Kumral et al., 2005). Moreover, studies have also shown that EPO can improve the prognosis of the nervous system in patients with acute ischemic stroke (Tsai et al., 2015) and reduce brain edema responses after experimental traumatic brain injury (Blixt et al., 2018). Yet, other researchers have shown that EPO does not reduce neurological dysfunction and does not have the protective effect of nerve cells in treatments of traumatic brain injury (Nichol et al., 2015). 

Mechanistically, the exact mechanism of action remains unclear. Some studies have suggested that the protective effect of EPO on nerve cells is the result of the joint action of multiple genes. In this study, the hypoxia model of U251 Cell glioma line was constructed by the CoCl_2_ method. The effect of EPO on the proliferation of the U251 Cell glioma line in the hypoxia model was explored, the expression changes in the transcriptome were screened by high-throughput sequencing technology, and the differentially expressed genes PI3K, IKKα, and CDK1 were verified. Then, PI3K inhibitor LY294002 and CDK1 interference RNA methods were employed to explore the effect of EPO on the PI3K-IKKα-CDK1 signaling pathway of U251 cell glioma line under hypoxia, so as to an provide experimental basis for studying the neuroprotective mechanism of EPO in brain injury caused by neonatal asphyxia.

## Material and Methods

### CCK-8 assay to detect the effect of CoCl_2_ on U251 cell proliferation

The U251 cell glioma line was obtained from National Collection of Authenticated Cell Cultures,RRID:CVCL_0021, China, 2018. Cells were divided into three groups: blank control group, MgCl_2_ group, and CoCl_2_ group. Briefly, 2000 cells per well were inoculated into 96-well plates, with three replicates in each group. The blank control group was cultured in a normal culture medium without additional treatment; the MgCl_2_ group was cultured in a normal culture medium containing 400 μmol/L MgCl_2_, and the CoCl_2_ group in 400 μmol/L CoCl_2_ (Sigma, USA) culture medium. After 24 hours, 20 μl of CCK-8 solution (Yeasen Biotechnology, Shanghai, China) was added to each well, and the culture plate was incubated in the incubator for an additional 2 hours. The light absorption value of each well was measured on the microplate reader (OD450 nm), the results were recorded, and the cell viability value was calculated. Taking the CoCl_2_ group as an example, the cell viability value% = [OD (CoCl_2_ group) - OD (blank)] / [OD (blank control group) - OD (blank)] × 100%. 

### Quantitative real-time RT-PCR (qPCR) to determine the expression of HIF-1α mRNA in U251 cells of each group after adding CoC1_2_

The cells of the blank control group and CoCl_2_ group were extracted with an RNA extraction kit (TIANGEN Company, Beijing, China) and reversely transcribed into cDNA using an RNA reverse transcription kit (TOYOBO, Japan). qPCR was carried out according to the instructions of TOYOBO Company. The reaction volume was 20 μL, including 10 μL Master Mix, 1 μL cDNA, 1 μL Forward Primer, 1 μL Reverse Primer, and 7 μL deionized water. Then, a relative quantitative (RQ) value (RQ = 2^-ΔΔCT^) was calculated, which represents the relative expression level of the genes. The following primer sequences were used:

GAPDH: (5’-3’) Forward primer GGAAGGACTCATGAC CACAGT 

 Reverse primer GGCAGGTTTTTCTAGAC GGC 

HIF-1α: (5’-3’) Forward primer GGCAGCAACGACA CAGAAAC

 Reverse primer TGCAGGGTCAGCAC TACTTC

### Western blot (WB) to detect the expression of HIF-1α protein in CoC1_2_ group and blank control group cells

Total protein was extracted by total protein extraction kit (KeyGEN BioTECH, Jiangsu, China), the concentration was assessed and the samples were boiled for 5 min. Then, proteins were separated by electrophoresis and transferred to the PVDF membrane, and blocked with a blocking solution (Sigma, USA) at room temperature for two hours. Next, samples were incubated with anti-HIF-1α (1:500, Sangon Biotech, Shanghai, China) and anti-ACTB (1:500, Sangon Biotech, Shanghai, China) antibody overnight at 4 °C, and then with anti-rabbit IgG, HRP-linked antibody (1:1000, CST, USA) at room temperature for 1 hour. Finally, samples were analyzed using a gel imager. 

### The effect of EPO on U251 cell proliferation under hypoxia by CCK-8 assay

The cells were divided into three groups. The blank control group was cultured in culture medium without additional treatment for 48h; the CoCl_2_ group was cultured in a normal culture medium 400 μmol/L CoCl_2_ for 48 h; the EPO group was cultured in a normal culture medium with 400 μmol/L CoCl_2_ for 24 h, and then with 75I U/ml EPO (Beijing Four Rings, Beijing, China) for additional 24 h. The results were recorded, and the cell viability value was calculated.

### The effect of EPO on the transcriptome of U251 cells under hypoxia by transcriptome sequencing

The CoCl_2_ group and EPO group were selected with three repeats. Then, a library was established. After the quality inspection, the samples were sequenced, and then the optical signals were converted into sequencing peaks through computer software to obtain the sequence information of test pieces. Expression analysis values of the transcripts were computed by StringTie. Genes were considered as significant differentially expressed at p-value <0.05. Gene expression differences were visualized by scatter heat map and volcano plot. Functional analysis of differentially expressed genes by Gene Ontology (GO) and KEGG was performed to identify which DEGs were significantly enriched in GO terms or metabolic pathways. qPCR to determine the expression of significantly up-regulated genes included PI3K, IKKα, and CDK1 in U251 cells of each group.The following primer sequences were used:

β-actin: (5’-3’) Forward primer TAGTTGCGTTACAC CCTTTCTTG 

Reverse primer TCACCTTCACCGTTC CAGTTT 

PI3K: (5’-3’) Forward primer TCTGGAAAAATGGCTTT GAATC

Reverse primer CTGGGAACTTTACCA CACTGCT

IKK-α: (5’-3’) Forward primer GAACGTCTGTCTGTAC CAGCATC

Reverse primer TCCTCCAGAACAGTAT TCCATTG

CDK1: (5’-3’) Forward primer GTCAGTCTTCAGGAT GTGCTTATG

Reverse primer CATGTACTGACCAG GAGGGATAG

### The expression of PIK3, IKKα, and CDK1 by WB method

Total protein was extracted by a total protein extraction kit. The concentration was assessed and the samples were boiled in a blank control group, CoC1_2_ group, and EPO group cells. Then, it was separated by electrophoresis and transferred to the PVDF membrane, and blocked with blocking solution. Next, samples were incubated with anti-CDK1 (1:300, Sangon Biotech), anti-IKKα, anti-PIK3 (1:500, Sangon Biotech, Shanghai,China), and PI3 Kinase p110α (1:1000, CST, USA) antibody overnight at 4°C, and then with anti-rabbit IgG, HRP-linked antibody (1:1000, CST, USA) at room temperature for 1 hour. The samples were analyzed using a gel imager.

### The IC50 concentration of inhibitor LY294002 by CCK-8 assay

Briefly, 2,000 cells were plated in 96-well plates and incubated for 24 h. Cells were then exposed to gradually increased concentration ((0.01, 0.1, 1, 3, 10, 30, 100, 300, and 1000 μmol/L) of LY294002 (MCE, USA,DMSO was configured as a 50 mmol/L storage solution) for 24 h. The absorbance at 450 nm was then recorded, and the cell viability value and IC50 concentration were calculated.

### CCK-8 assay to detect the intervention effect of EPO on U251 cells under hypoxia through PI3K signaling pathway

The experiment was divided into six groups: blank control group, CoC1_2_ (C group), CoC1_2_ + EPO (C+E group), CoC1_2_ + DMSO (0.1% DMSO, C+D group), CoC1_2_ + LY294002 group (18.31 μmol/L LY294002, C+ L group), CoC1_2_ + LY294002+ EPO group (18.31 μmol/L LY294002, 75 IU/ml EPO, C + L + E group). CCK-8 assay was used to detect the results and calculate the cell viability of each group at the same time.

### Effect of inhibition of PI3K expression on IKKα and CDK1 transcription level under hypoxia

qPCR was used to detect the relative expression of genes in the CoC1_2_ group (C group), CoC1_2_ + DMSO group (C+D group), and CoC1_2_ + LY294002 group (C+L group). 

### Construction and identification of eukaryotic expression vector of CDK1 interfering RNA

CDK1 interfering RNA eukaryotic expression vector and NC control vector were constructed and sequenced. U251 cells were transfected with Lipofectamine 2000 (Invitrogen, USA), and the expression of green fluorescent protein in each group was observed under an inverted fluorescence microscope. After successful transfection, 400 μmol/L CoCl_2_ was added to the culture for 24h, after which cells were divided into NC + CoC1_2_ group (NC group), Sh-CDK1-1+CoC1_2_ (Sh-1 group), Sh-CDK1-2+CoC1_2_ (Sh-2 group), and Sh-CDK1-1+CoC1_2_ (Sh-3 group). qPCR was used to detect the interference effect of the NC group and three CDK1 interfering RNA eukaryotic expression vectors under a hypoxia environment. The following short hairpin DNA design target sequence for constructing CDK1 vector was used:

Sh-CDK1-1: 5’- AAGGAACTTCGTCATCCAAATAT-3’

Sh-CDK1-2: 5’- AAGAGTTCTTCACAGAGACTTAA -3’

Sh-CDK1-3: 5’- TGGAGTATAGGCACCATATTTGC -3’

### CCK-8 assay was used to detect the intervention effect of EPO on U251 cells under hypoxia through CDK1

The experiment was divided into six groups: blank control group, CoC1_2_ (C group), CoC1_2_ + EPO (C+E group), CoC1_2_ + NC (C+NC group), CoC1_2_ + Sh-CDK1-2 group (C+ S group), CoC1_2_ +Sh-CDK1-2+EPO (C + S + E group). CCK8 assay was used to detect, record the results and calculate the cell viability of each group at the same time.

### Statistical analysis

Data of comparing the two groups was analyzed by *t*-test provided by GraphPad Prism 7 software. Data of comparing more than 3 groups was analyzed by one-way analysis of variance (Newman-Keuls:compare all pairs of columns) provided by GraphPad Prism 7 software. All P-values were two-sided, and P<0.05 was considered as the statistically significant difference.

## Results

### The effect of CoCl_2_ on U251 cell proliferation

**Figure 1 -:**
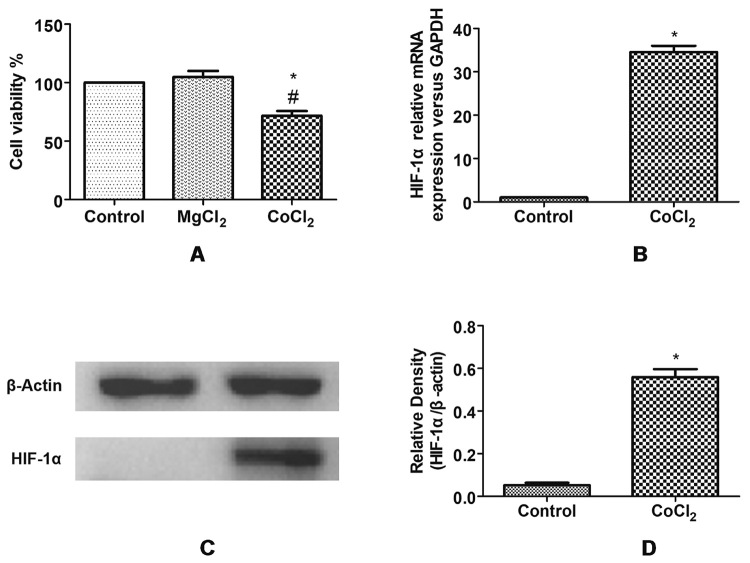
Establishment and evaluation of hypoxia model of U251 cells using CoCl_2_. **(A)** CCK-8 assay to detect the effect of CoCl_2_ on cell proliferation; * P<0.05 vs. the blank control group, # P<0.05 vs. the MgCl_2_ group, Newman-Keuls multiple comparisons test. **(B)** qPCR was used to determine the expression of HIF-1α mRNA in U251 cells of each group after adding CoC1_2_; *P<0.05 vs. the blank control group, T-test. **(C)** WB method to detect the expression of HIF-1α protein after adding CoC1_2._ **(D)** *P<0.05 vs. the blank control group, T-test.

There was no difference in cell proliferation in the MgCl_2_ group and the blank control group (P>0.05), while the cell proliferation level in the CoCl_2_ group was significantly reduced (P<0.05). The results are shown in Figure 1A.

### The effect of CoCl_2_ on hypoxia-inducible factor HIF-1α

Compared with the blank control group, the mRNA transcription level (Figure 1B) and protein expression level (Figure 1C, D) of HIF-1α in CoCl_2_ group cells were significantly reduced (P<0.05), suggesting that the cell hypoxia model was successfully constructed by CoCl_2_.

### The effect of EPO on cell transcriptome under hypoxia

**Figure 2 -:**
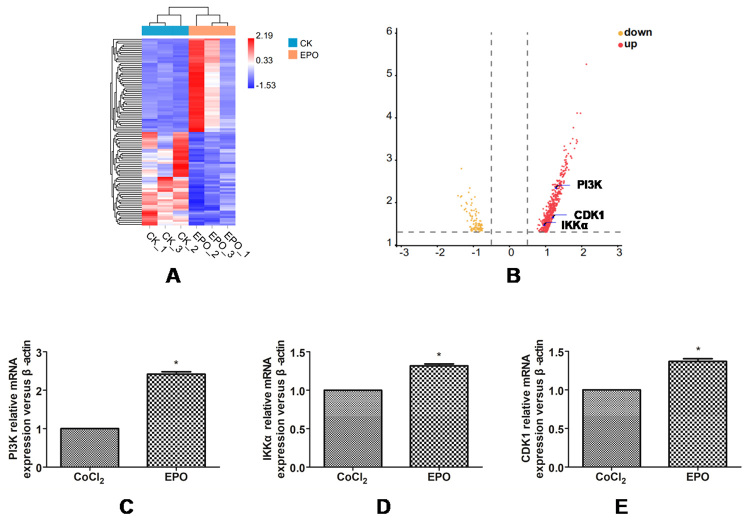
The effect of EPO on cell transcriptome under hypoxia by high throughput sequencing. **(A)** The heat map,50 genes of up-regulation and 50 genes of down-regulation were selected for visualization, and the genes were sorted according to p value significance. **(B)** The volcano plot is mapped according to log2FC>0.5, p<0.05, marking three target genes. (**C-E)** mRNA transcription levels of PIK3, IKKα and CDK1 were verified by qPCR method. * P<0.05 vs. the CoC1_2_ group, t-test.

The gene-level of the CoC1_2_ group (CK_1,2,3) and CoC1_2_ + EPO (EPO_1,2,3) group was analyzed by HTSeq software. Compared with the CoC1_2_ group, the gene expression level of the CoC1_2_ + EPO group showed 558 up-regulated genes and 98 down-regulated genes. According to the sequencing results (KEGG pathway analysis identifies significantly enriched in PI3K-AKT signaling pathways and cell cycle signaling pathways.PI3K, IKKα and CDK1 were considered as significant differentially expressed and up-regulated, P-value <0.05,log2FC>0.5.) and some studies, PI3K, IKKα and CDK1 were associated with the ability of cells to proliferate (Fernandez-Majada et al., 2007; Tse et al., 2017; Li et al., 2019; Jin et al., 2021). At present, studies have investigated the effect of EPO on its transcription level under CoC1_2_-induced hypoxia, l*et al.*one the effect on the PI3K-IKKα-CDK1 signaling pathway. The most significantly up-regulated genes included PI3K, IKKα, and CDK1 (Figure 2A, B). The result of qPCR showed that compared with the CoCl_2_ group, mRNA transcription levels of CDK1 and IKKα genes in the EPO group increased (P<0.05) (Figure 2C-E), which was consistent with the results of high-throughput sequencing.

### The effect of EPO on the proliferation of U251 cells under hypoxia

**Figure 3 -:**
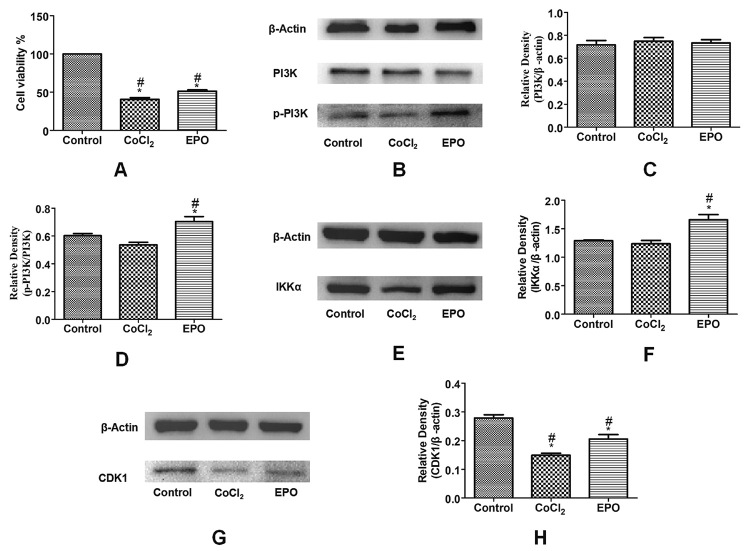
Effect of EPO on U251 cells under hypoxia. **(A)** The effect of EPO on the proliferation of U251 cells under hypoxia was determined by CCK-8 assay. ^#^P<0.05 vs. the blank control group.* P<0.05 vs. the CoC1_2_ group. (**B-H)** Expression of PIK3, p-PIK3,IKKα and CDK1 verified by WB method. ^#^ P<0.05 vs. the blank control group.* P<0.05 vs. the CoC1_2_ group, Newman-Keuls multiple comparisons test.

The results (Figure 3A) showed that the proliferation ability of the CoCl_2_ group and EPO group decreased compared with the blank control group, and the proliferation ability of the EPO group significantly increased compared with the CoCl_2_ group (P<0.05), thus indicating that 75IU EPO could promote cell growth under hypoxia induced by CoCl_2_.

### Expression of PIK3, IKKα, and CDK1 genes

The results showed that compared with the CoCl_2_ group, the expression levels of CDK1 and IKK**α** genes in the EPO group increased (P<0.05) (Figure 3E-H), which was consistent with the results of high-throughput sequencing. However, there was no difference in the expression level of PI3K among the three groups. Therefore, the phosphorylated PI3K (p-PI3K) was continuously detected, and the value of p-PI3K/PI3K in the EPO group was higher than that in the CoCl_2_ group (P<0.05) (Figure 3B-D), which again, was consistent with the results of high-throughput sequencing. This indicates that 75 IU EPO may promote cell growth by acting on the PI3K-IKKα-CDK1 signaling pathway under hypoxia induced by CoCl_2_. 

### The intervention effect of EPO on U251 cells under hypoxia through PI3K signaling pathway

**Figure 4 -:**
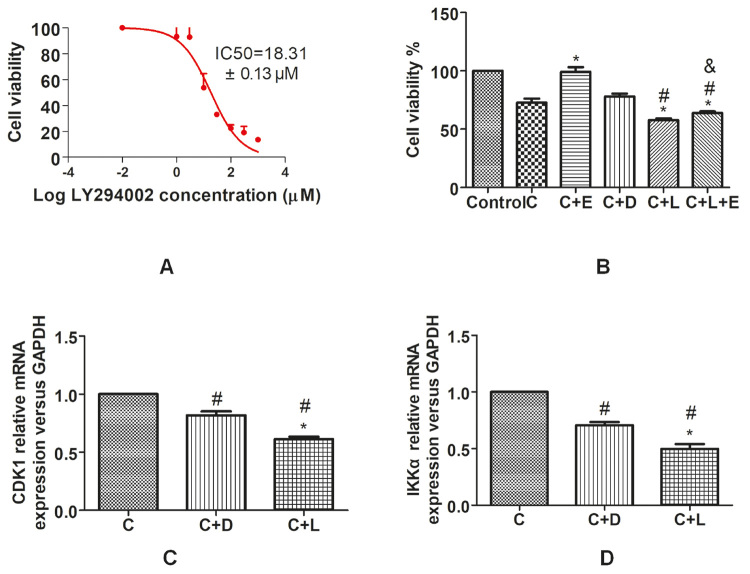
The intervention effect of EPO on U251 cells under hypoxia through PI3K signaling pathway. **(A)** The IC50 concentration of the inhibitor LY294002. **(B)** The cell viability of effect of EPO on U251 cells. ^#^P<0.05 vs. the CoC1_2_+DMSO group.* P<0.05 vs. the CoC1_2_ group and &P<0.05 vs. the CoC1_2_+EPO group. (**C, D)** The effect of inhibition of PI3K expression on IKK ***α*** and CDK1 transcription level under hypoxia.#P<0.05 vs. the CoC1_2_ group.* P<0.05 vs. the CoC1_2_+DMSO group, Newman-Keuls multiple comparisons test.

The inhibitor LY294002 showed an obvious inhibitory effect on U251 cells, and the IC50 concentration was 18.31 μmol/L (Figure 4A). After the inhibitor LY294002 acted on the cells under hypoxia, the effect of EPO on the cell proliferation ability was determined (Figure 4B). After the same time of culture, the cell proliferation level of the CoC1_2_+EPO group increased, while the cell proliferation level of the the CoC1_2_+LY294002 group and the CoC1_2_+LY294002+EPO group decreased compared with the CoC1_2_ group (all P<0.05). Compared with the CoC1_2_+EPO group, the cell proliferation level in the CoC1_2_+LY294002+EPO group decreased (P<0.05). Compared with the CoC1_2_+DMSO group, the cell proliferation level ofCoC1_2_+LY294002 group and the CoC1_2_+LY294002+EPO group decreased (P<0.05), while the cell proliferation level of the CoC1_2_+LY294002 group and CoC1_2_+LY294002+EPO group showed no difference. This indicated that EPO could promote cell growth under hypoxia induced by CoCl_2_, but when PI3K was inhibited, EPO also lost its promoting effect on cell growth. These results suggested that EPO promotes cell growth through PI3K signaling pathway under hypoxia induced by CoCl_2_.

### Effect of inhibition of PI3K expression on IKKα and CDK1 transcription level under hypoxia

The relative expression levels of IKKα and CDK1 in the CoC1_2_ group, CoC1_2_+DMSO group, and CoC1_2_+LY294002 group were detected by qPCR (Figure 4C, D). Compared with the CoC1_2_ group, the expression levels of IKKα and CDK1 mRNA in the CoC1_2_+DMSO group and CoC1_2_+LY294002 group decreased, and the expression levels of IKKα and CDK1 mRNA in the CoC1_2_+LY294002 group decreased compared with the CoC1_2_+DMSO group (all P<0.05), which suggested that IKKα, CDK1, and PI3K are induced by CoCl_2_, and IKKα and CDK1 are located downstream of the PI3K signaling pathway.

### Construction and identification of eukaryotic expression vector of CDK1 interfering RNA

**Figure 5 -:**
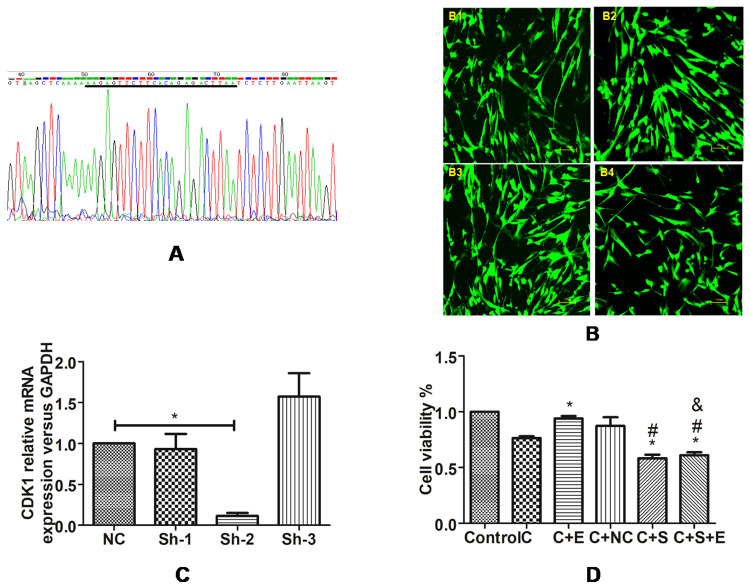
Intervention of EPO on U251 cells under hypoxia through CDK1. **(A)** Partial sequencing of Sh-CDK1-2 (The part marked by black line is target sequence). **(B)** The expression of green fluorescent protein in each group: B-1, NC group; B-2, Sh-CDK1-1 group; B-3, Sh-CDK1-2 group; B-4, Sh-CDK1-3 group, 400*. **(C)** The interference effect of CDK1 interfering RNA eukaryotic expression vector under hypoxia environment.* P<0.05 vs. the NC group. **(D)** CCK-8 assay was used to detect the intervention effect of EPO on cells under hypoxia through CDK1.* P<0.05 vs. the CoC1_2_ group; ^#^ P<0.05 vs. the CoC1_2_+NC group and & P<0.05 vs. the CoC1_2_+EPO group, Newman-Keuls multiple comparisons test.

The results of base sequencing and identification of the CDK1 interfering RNA eukaryotic expression vector showed the same designed sequence and no gene abnormalities such as mutation, deletion, and insertion (taking Sh-CDK1-2 as an example, see Figure 5A). Twenty-four hours after transfection, all cells in each group were observed under an inverted fluorescence microscope, and green fluorescent protein was expressed (Figure 5B).

### The interference effect of CDK1 interfering RNA eukaryotic expression vector under hypoxia environment

qPCR detection of the interference effect of each group of interference vectors under hypoxia environment revealed (Figure 5C) that the mRNA expression level of CDK1 in the Sh-2 group decreased compared with the NC group (all P<0.05), while the mRNA expression level of the Sh-1 group and Sh-3 group showed no statistical difference (all P>0.05). Therefore, the Sh-CDK1-2 eukaryotic expression vector was selected for subsequent experiments.

### CCK-8 assay was used to detect the intervention effect of EPO on cells under hypoxia through CDK1

After culture for the same time, results of CCK-8 assay showed that (Figure 5D) compared with the CoC1_2_ group, the proliferation level of cells in the CoC1_2_+Sh-2 group decreased (P<0.05),while the cell proliferation level of CoC1_2_ group and CoC1_2_+NC group showed no difference (P>0.05). This data suggests that Sh-CDK1-2 eukaryotic expression vector had an obvious inhibitory effect on U251 cells. Compared with the CoC1_2_ group, the proliferation level of cells in the CoC1_2_+EPO group increased, while the proliferation level of cells in the CoC1_2_+Sh-2+EPO group decreased (all P<0.05), while the cell proliferation level of CoC1_2_+ Sh-2 group and CoC1_2_+Sh-2+EPO group showed no difference (P>0.05).These data suggests that when CDK1 expression was inhibited, EPO lost its promoting effect on cell growth. 

## Discussion

Neonatal asphyxia often leads to neonatal hypoxic-ischemic encephalopathy, cerebral palsy, intellectual disability, etc., and is a common cause of neonatal death worldwide. Early intervention is an effective method for preventing and treating neurological sequelae. Although the beneficial effects of EPO in neuroprotection have been proved in some studies on rodents (Yuan et al., 2015; Dmytriyeva et al., 2016), its clinical experiments and basic mechanism are still controversial (Schreiber et al., 2017). In this study, we explored the molecular mechanism of EPO’s neuroprotective effect on neonatal asphyxia. Our data suggested that EPO promotes the proliferation of U251 cells by activating the PI3K-IKKα-CDK1 signaling pathway in a hypoxic environment and has a protective effect on hypoxic glial cells.

CoCl_2_ is a commonly used chemical hypoxia simulator, which causes cell hypoxia. CoCl_2_ simulates the hypoxia microenvironment *in vivo* by promoting the expression of HIF-1 (Byrne et al., 2014) and stimulating ROS production (Lin et al., 2017). HIF is the main transcription factor regulating hypoxia (Myllyharju, 2013). The activation of HIF-1 is an efficient and rapid hypoxia response mechanism in the human body (Kaluz et al., 2008). HIF-1, which is composed of two subunits, α and β, can participate in glycolysis metabolism and erythropoiesis and is relatively stable during hypoxia (Semenza, 2014). Some studies have shown that HIF-1α in newborn rats significantly increases during hypoxia (Kletkiewicz et al., 2018). Chu et al. (2016) used CoCl_2_ to establish a hypoxia model of breast cancer cells and found that the expression of HIF-1α mRNA was increased in those cells. In this study, human glioma U251 cells were treated with 400 μM CoCl_2_. Compared with the blank control group and MgCl_2_ group, the cell proliferation level decreased, and the expression of HIF-1α mRNA and protein increased in a CoCl_2_ group, suggesting that a hypoxia cell model was successfully established. 

Over recent years, many studies have reported on the role of EPO in neuroprotection. One study found that after EPO was used to treat neonatal rats with hippocampal injury model, neurobehavioral performance was significantly improved, and neuronal death induced by hippocampal injury was avoided (Lan et al., 2016). Moreover, Cohrs et al. (2018) found that EPO promotes nerve regeneration potential in spinal cord injury and may participate in the development of other sequelae. Ren and colleagues found that EPO can reduce brain edema and improve spatial learning ability and memory in rats (Ren *et al.*, 2017). Blixt et al. (2018)*.* found that EPO can reduce traumatic cell edema by protecting the structure and functional characteristics of the blood-brain barrier after experimental craniocerebral trauma. In addition, Moransard and colleagues discovered that EPO alleviates the severity of experimental autoimmune encephalomyelitis by improving the survival of spinal cord neurons (Moransard *et al.*, 2017). Yet, research on the transcriptome level of EPO after acting on hypoxic glial cells has been rarely reported.

In this study, we investigated the transcription level of EPO on U251 cells under hypoxia. The experimental results showed that EPO could promote the proliferation of glial U251 cells under a hypoxia environment. High-throughput sequencing technology,GO and KEGG pathway analysis were used to screen up-regulated and significant differentially expressed genes PI3K, IKKα, CDK1 related to proliferation. WB verification results were consistent with high-throughput sequencing results, suggesting that EPO may promote glial cells’ growth under hypoxia by activating the PI3K-IKKα-CDK1 signaling pathway.

In this experiment, we further verified whether EPO could activate PI3K signaling pathway under hypoxia. The experimental results showed that EPO could promote cell proliferation and PI3K expression under hypoxia, while this effect was inhibited after adding LY294002 (PI3K inhibitor) to cell culture. Jia *et al.* (2014) used EPO to prevent neuronal apoptosis in Parkinson’s disease model. They found that EPO increased the expression of phosphorylated PI3K, while the PI3K inhibitor LY294002 significantly reversed EPO-dependent neuroprotection. Moreover, EPO could promote axon germination and GDF10 expression, which LY294002 could block (Li et al., 2019). All these experiments showed that EPO can exert a neuroprotective role through the PI3K signaling pathway, which is consistent with our results. However, PI3K signaling involves many genes. At present, no studies have investigated the effect of EPO on its transcription level under CoC1_2_-induced hypoxia, l*et al.*one the effect on the PI3K-IKKα-CDK1 signaling pathway. Our data show that the expression of IKKα and CDK1 increases with PI3K, while the expression of IKKα and CDK1 decreases when PI3K is inhibited, suggesting that IKKα and CDK1 are downstream of the PI3K pathway that can be activated by EPO.

Studies have shown that IKKα can specifically connect with the Notch target promoter, causing the release of chromatin SMRT, which activates the transcription of *hes1* or *hes5*, and activating cell proliferation by inhibiting cycle-dependent protein kinase inhibitor p27 (Fernandez-Majada et al., 2007). In keratinocyte differentiation, IKKα is a part of the Smad 2/3 signaling pathway and has a role in controlling the cell cycle. The regulation of the cell cycle by Smad 2/3 is combined with p53, and the phosphorylation of p53 is regulated by IKKα (Tse et al., 2017). Therefore, IKKα can promote and participate in cell proliferation in the cell cycle, which is consistent with the fact that EPO promoted cell proliferation through the PI3K-IKKα signaling pathway in the present study. However, no studies have examined EPO interference with CDK1 protein at the end of the cell cycle through PI3K-IKKα signaling pathway under CoC1_2_-induced hypoxia.

CDK1 is mainly responsible for entering the S phase from the G1/S restriction point in the cell cycle. After entering the S phase, CDK1 participates in the regulation of DNA replication and centrosome replication and promotes mitosis. The high expression of CDK1 can increase the expression of the maturation-promoting factor complex and promote cells to enter the M phase from the G2/M phase (Jin et al., 2021). This study showed that EPO could promote cell proliferation and CDK1 expression under hypoxia, but transfection of CDK1 interfering RNA eliminated the promoting effect of EPO on cell proliferation. This suggested that EPO can promote cell proliferation through CDK1 protein, further proving that EPO may promote glial cell growth under hypoxia by activating the PI3K-IKKα-CDK1 signaling pathway.

To sum up, our data suggested that EPO promotes the proliferation of U251 cells by activating the PI3K-IKKα-CDK1 signaling pathway, which further proved that EPO had a protective effect on hypoxic glial cells. These data provide a meaningful molecular basis for the role of EPO in the treatment of neonatal asphyxia brain injury. However, the U251 glioma cell line was derived from human gliomas, so this study had some limitations. We will use normal human glial cells to construct the model in further experiments.
